# Design, In Vitro Evaluation and In Vivo Biocompatibility of Additive Manufacturing Three-Dimensional Printing of β beta-Tricalcium Phosphate Scaffolds for Bone Regeneration

**DOI:** 10.3390/biomedicines12051049

**Published:** 2024-05-09

**Authors:** José Javier Llorente, Luis Junquera, Lorena Gallego, Marcos Pérez-Basterrechea, Luis Ignacio Suárez, Santiago Llorente

**Affiliations:** 1 Department of Orthopaedic Surgery, Ribera Povisa Hospital, 26211 Vigo, Spain; jjllorente@povisa.es; 2 Department of Surgery, University of Oviedo, 33006 Oviedo, Spain; junquera@uniovi.es; 3 Department of Oral and Maxillofacial Surgery, Central University Hospital, 33011 Oviedo, Spain; 4 Department of Oral and Maxillofacial Surgery, Cabueñes University Hospital, 33394 Gijón, Spain; 5 Cell Therapy and Regenerative Medicine Unit, Central University Hospital, 33011 Oviedo, Spain; marcos.perez@sespa.es; 6 Advanced Manufacturing Area, IDONIAL Technology Centre, 33203 Gijón, Spain; luisignacio.suarez@idonial.com; 7 Head and Neck Center Dr. Llorente, 33005 Oviedo, Spain; llorentesantiago@gmail.com

**Keywords:** 3D scaffold, bone regeneration, tissue engineering, 3D printing

## Abstract

The reconstruction of bone deficiencies remains a challenge due to the limitations of autologous bone grafting. The objective of this study is to evaluate the bone regeneration efficacy of additive manufacturing of tricalcium phosphate (TCP) implants using lithography-based ceramic manufacturing (LCM). LCM uses LithaBone TCP 300 slurry for 3D printing, producing cylindrical scaffolds. Four models of internal scaffold geometry were developed and compared. The in vitro studies included cell culture, differentiation, seeding, morphological studies and detection of early osteogenesis. The in vivo studies involved 42 Wistar rats divided into four groups (control, membrane, scaffold (TCP) and membrane with TCP). In each animal, unilateral right mandibular defects with a total thickness of 5 mm were surgically performed. The animals were sacrificed 3 and 6 months after surgery. Bone neoformation was evaluated by conventional histology, radiology, and micro-CT. Model A (spheres with intersecting and aligned arrays) showed higher penetration and interconnection. Histological and radiological analysis by micro-CT revealed increased bone formation in the grafted groups, especially when combined with a membrane. Our innovative 3D printing technology, combined with precise scaffold design and efficient cleaning, shows potential for bone regeneration. However, further refinement of the technique and long-term clinical studies are crucial to establish the safety and efficacy of these advanced 3D printed scaffolds in human patients.

## 1. Introduction

The satisfactory repair of bone defects of the craniofacial skeleton, resulting from trauma, tumor resection or inborn disorders, remains one of the main concerns of reconstructive surgeons. The use of autologous bone grafts is currently considered the gold standard for bone repair and reconstruction. However, limited sources and complications, such as pain, infection, fractures, and possible nerve injuries after surgery, restrict its clinical application [1,2]. On the other hand, applying tissue-engineering principles to skeletal reconstruction may enhance both morphological and functional results while overcoming these main disadvantages. Scaffolds play an essential role in supporting bone regeneration in bone tissue engineering. In order to obtain an ideal bone substitute implant material, high porosity, and good cytocompatibility are critical factors [2,3].

The ideal scaffold should be osteoconductive and provide adequate environmental interactions. Regarding osteoconductive microarchitecture, studies have demonstrated that bone substitutes comprising concave pores induce significantly more bone tissue growth than smooth surfaces [4], and bone formation is enhanced by the concavities on the surface of calcium phosphate-based bone substitutes [5]. Other important microarchitectural feature widely studied is the optimal pore diameter. A bone substitute pore diameter of 0.3–0.5 mm has long been considered as optimal for bone formation and osteoconduction [6,7,8,9]. There is only one in vivo study with undefined random pore locations and connections between pores, to our knowledge, suggesting that bone growth is comparable in pores of 0.5 mm to 1.2 mm [10]. Conventional cell-based tissue engineering constructs appear to be limited in the repair of large bone defects due to the lack of a mature vascular network, which is essential to ensure an adequate supply of nutrients and oxygen to adherent/migrated cells beyond 200 μm from the closest blood capillary [11,12]. Three-dimensional printing (3DP) is an additive manufacturing technique that produces physical models by adding materials layer by layer based on computer-aided design, allowing for the cost-effective and fast manufacturing of a variety of items [13]. The advent of 3D-printed metal, ceramic, polymer, and composite scaffolds that mimic the complex geometries and mechanical properties of native tissue has greatly accelerated the reconstruction of large bone defects in humans and animals [11].

New ceramic additive manufacturing methodologies enable full control of pore size and distribution, even the addition of antibacterial coatings with both resistance and bacterial killing properties, which exhibit excellent performance in antibacterial adhesion and prevention of biofilm formation. Thus, this appears to be the method of choice for testing the characteristics of an optimal bone substitute [14,15]. Bone tissue engineering scaffolds with mesenchymal stem cells (MSCs), co-cultured on 3D-printed composite bioactive ceramic scaffolds, have also been shown to promote osteogenesis/angiogenesis [16]. Therefore, the robocasting of customized β-tricalcium phosphate (β-TCP) scaffolds, often combined with cell and/or growth factors, could successfully repair large bone defects [11,16]. The ceramic 3D printing market is expected to reach $4.8 billion by 2030 [8].

The so-called lithography-based ceramic manufacturing (LCM) represents a novel alternative within the field of 3D printing for the processing of ceramic materials. The technique derives from Digital Light Processing (DLP) technology. In recent years, its development has made it possible to personalize the manufacturing process, but it also requires verification of the synthetic materials traditionally used in the bone regeneration process. These materials could lose their bone regeneration properties during the additive manufacturing process.

The aim of this project was to test the feasibility of additive manufacturing of tricalcium phosphate implants using lithography-based ceramic manufacturing (LCM) with a porosity suitable for bone growth and to ensure that this process did not affect the well-established osteoconductive properties of tricalcium phosphate. Once the parts had been produced, they were tested in an animal experimental model.

## 2. Materials and Methods

We used LithaBone TCP 300 [Ca_3_(PO_4)2]_ (Lithoz GmbH, Vienna, Austria) as a photosensitive slurry for 3D printing. This slurry consisted of tricalcium phosphate powder with particles ranging from 5 to 30 µm, together with undeveloped components, such as acrylate-based monomer, photoinitiator, light absorber, and organic solvent. The CeraFab 8500 printer (Lithoz, Vienna, Austria) was used to solidify the slurry layer by layer, resulting in a green part with a resolution of 25 µm in layer thickness and 50 µm in the x/y plane. Afterwards, the green parts were removed, cleaned, and subjected to a heat treatment process to remove the solvent, decompose the polymer bonding agent and sinter (densify) the samples, with a final sintering step of 3 h at 1200 °C.

### 2.1. Design and Fabrication of Scaffolds

**Figure 1:**
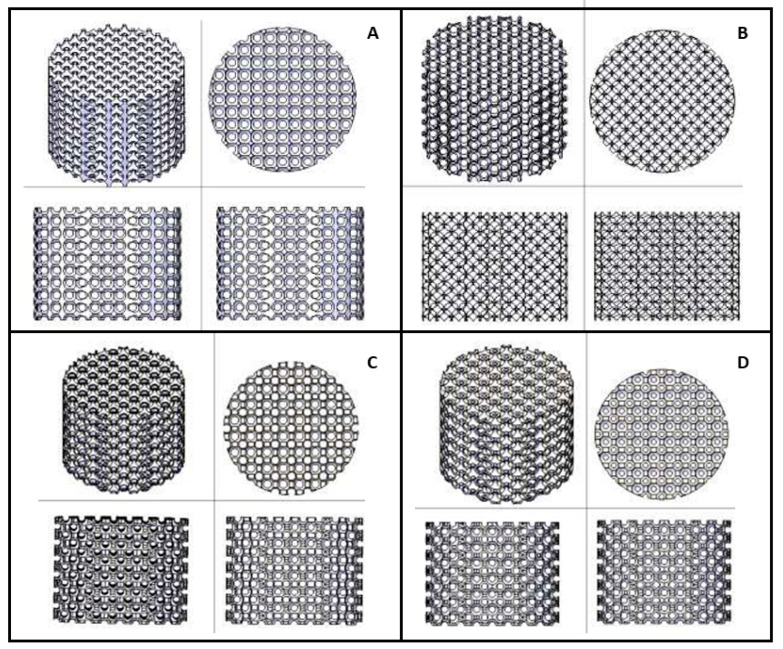
Internal geometry of the four studied models. (**A**) Model A: spheres with intersection; aligned matrices, empty volume: 24.52%. (**B**) Model B: spheres with intersection; matrices not aligned, empty volume: 6.21%. (**C**) Model C: separate spheres, matrices not aligned, joined with 0.3 mm diameter cylindrical channel, empty volume: 17.42%. (**D**) Model D: separate spheres, matrices not aligned, joined with cylindrical channels of diameter 0.2 mm, empty volume: 23.54%.

SolidWorks (Dassault Systèmes SolidWorks Corporation, Waltham, MA, USA) was used for designing cylindrical parts with a diameter of 5 mm and a height of 2 mm to replicate the intended bone defect. Spherical pores of 500 microns were interconnected, establishing an overlapping rate of pores between 75% and 80% of the total implant volume. The interconnections of the spheres could be established by intersections between the spheres or by increasing the spacing and creating intercommunicating cylindrical channels. Four internal scaffold geometries (models A–D) were developed for comparison (Figure 1). The arrangement of the spherical voids, as well as the dimensions and respective emptying volumes, were controlled: model A: spheres with intersection, aligned matrices, empty volume: 24.52%; model B: spheres with intersection, matrices not aligned, empty volume: 6.21%; model C: separate spheres, matrices not aligned, joined with a 0.3 mm diameter cylindrical channel, empty volume: 17.42%; model D: separate spheres, matrices not aligned, joined with cylindrical channels of diameter 0.2 mm, empty volume: 23.54%. After printing, the pieces underwent an ultrasonic bath cleaning in an organic solvent provided by the material supplier, and the quality and resolution of the internal mesh were compared.

### 2.2. Mechanical Testing

The tensile tests were carried out using the dynamo-mechanical analysis (DMA) model RSA3 equipment from TA. The equipment has a servo-mechanical motor system and a 35 N load cell that allow both static and dynamic tests to be carried out. It also has a controlled temperature chamber, which allows tests to be carried out in conditions other than the environment. Given the capacity of the load cell, it is calibrated using a set of approved masses through the calibration application included in the equipment software (TA ORCHESTRATOR7.2.0.4., New Castle, DE, USA). The equipment was calibrated prior to carrying out the tests. The tensile tests were carried out under ambient laboratory conditions and using a displacement speed of 1 mm/min, a speed that was considered slow enough to guarantee quasistatic test conditions. The stress applied ranged from 80 to 300 Pa. Scaffolds were used in their dried state.

### 2.3. In Vitro Study

#### 2.3.1. Cell Cultures

The adipose tissue is an important source of mesenchymal stromal cells (AD-MSC). A total of 1 cm^2^ of subcutaneous adipose tissue was collected from dorsal interscapular fat of adult male Wistar rats and transferred into a tissue collection recipient with Dulbeccos’s modified Eagle’s medium (DMEM, Gibco, Paisley, UK) containing 10% (*v*/*v*) fetal bovine serum (FBS, Gibco) and 0.4% (*v*/*v*) penicillin–streptomycin (Gibco). Once in the lab, the sample was processed under sterile conditions: the sample was washed three times in DMEM, placed in a Petri dish and minced into fragments of 2–3 mm in thickness. Then, tissue fragments were placed into a 50 mL tube with 0.1% collagenase I (Sigma-Aldrich, Madrid, Spain) in DMEM (Gibco), and the suspension was agitated and digested for 1 h at 37 °C. Digestion was stopped by adding FBS (Gibco) to DMEM, and the sample was filtered through a 40 µm cell strainer (Falcon, Durham, NC, USA). Cell suspension was then centrifuged at 500× *g* for 10 min, and the resultant pellet was resuspended in a complete medium: DMEM (Gibco), 20% FBS, 0.2% (*v*/*v*) penicillin–streptomycin (Gibco) and cultured into 25-cm^2^ polystyrene flasks (Cultek, Madrid, Spain), at 37 °C, in a humidified atmosphere of 5% CO_2_. The medium was replaced every 2–3 days. When the monolayer of adherent cells reached 80% confluence, cells were trypsinized and subcultured. AD-MSCs were used at passages 3–4.

#### 2.3.2. Differentiation of AD-MSC into Osteogenic Cells (AD-MSCOs)

Osteogenic differentiation was achieved with an osteogenic culture medium: DMEM supplemented with 10 nM dexamethasone, 10 mM b-glycerophosphate, 0.28 mM ascorbic acid (all from Sigma, St. Louis, MO, USA), and 10% human serum. The culture medium was changed two times per week. The cells were maintained for 1 month at 37 °C in a humidified atmosphere of 5% CO_2_. 

#### 2.3.3. Cell Seeding

AD-MSC (both osteogenic-differentiated and non-differentiated) were seeded separately over a scaffold in a 12-well dish and incubated for 3 h to 7 days at 37 °C and 5% CO_2_ prior to SEM study.

#### 2.3.4. Morphological Studies

The structural characteristics of the scaffold were examined by scanning electron microscopy (SEM). The sample prepared as previously described was fixed with a 2% phosphate-buffered 0.1 M glutaraldehyde, buffered with 2% phosphate for 12 h. The fixed samples were then dehydrated in a graded acetone series (30, 50, 70, 90, 90, 100%) and then critical point dried using CO_2_ (Baltec CDP 030 critical point dryer). The samples were coated with gold by sputtering and examined with a scanning electron microscope (JEOL JSM 6100, Tokyo, Japan). Average pore diameters were quantified by image analysis using SEM digital pictures of sectioned samples.

To assess the biocompatibility of the scaffold and the cells, the structural features of the scaffold alone and with AD-MSC were observed by SEM. A microanalysis of phosphorus and calcium was performed in different zones of the scaffold both without cells and with cells using a digitized energy-dispersive X-ray (EDX) image analysis system (EOX, Microanalysis, INCA Energy, Oxford Instruments, Concord, MA, USA).

#### 2.3.5. Detection of Early Osteogenesis

To detect early osteogenesis and confirm the osteoblastic nature of the differentiated cells, alkaline phosphatase staining was performed (Sigma Fast BCIP/Nbt, Barcelona, Spain).

### 2.4. In Vivo Studies

#### 2.4.1. Surgical Procedure

This study adhered to the ARRIVE guidelines and was conducted in compliance with the EU Directive 2010/63EU and national guidelines for the care and use of laboratory animals. Experimental protocols received approval from the Committee for Animal Care and Handling of the University of Oviedo (MaxiloprintIDE/2018/571). Animals were earmarked for identification, housed in facilities with controlled light, humidity, and temperature, and provided food and water ad libitum. Sample size estimation considered previous studies by our team [2] and the minimum number of animals required for significant conclusions, accounting for potential postoperative incidences.

**Figure 2:**
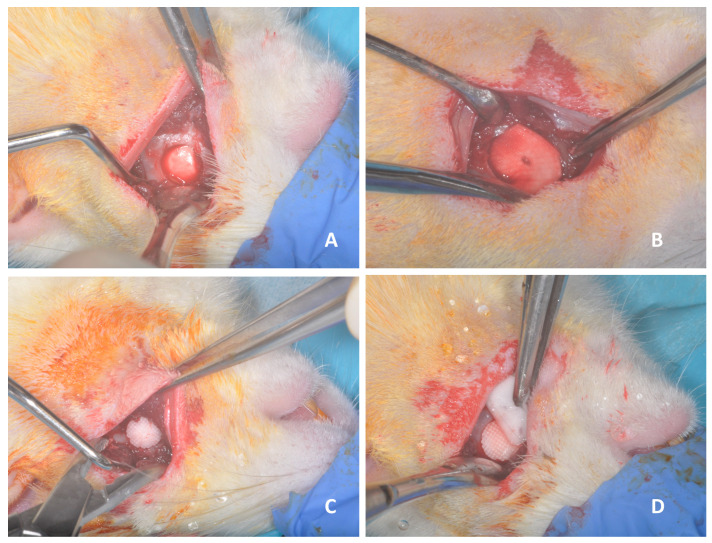
Images of the surgical procedure showing the 5 mm rounded bone defect made in hemimandible. (**A**) Control group: mandibular defect without graft or membrane. (**B**) Membrane group: mandibular defect covered with a resorbable collagen membrane. (**C**) Scaffold group: mandibular defect grafted with tricalcium phosphate implants. (**D**) Scaffold + membrane group: defects filled with tricalcium phosphate implants and covered with membrane.

Forty-two adult female Wistar rats weighing between 400–450 grams were selected and divided into four groups based on the implant used to fill the experimental defects. Control group control (group C): mandibular defect without graft or membrane (n = 6). Membrane group (group M): mandibular defect covered with a resorbable collagen membrane (Zimmer Biomet Dental, Warsaw, IN, USA) on the inner and outer surface of the ascending ramus of the mandible (n = 7). Scaffold group (group S): mandibular defect grafted with tricalcium phosphate implants developed by 3D printing (n = 8). Scaffold with membrane group (group SM): defects filled with tricalcium phosphate implants and covered with the same membrane (n = 9) (Figure 2).

Animals were anesthetized with an intramuscular injection of sodium pentobarbital (0.038 mg/g) and xylazine hydrochloride (0.075 mg/g). A right submandibular skin incision was made, and the mandibular periosteum was carefully dissected. With a trephine drill (Surgimedic, Asturias, Spain) irrigated with a 0.9% saline solution, unilateral right mandibular defects with a total thickness of 5 mm were surgically created in each animal. All surgeries were performed by a single surgeon (SLl). The selection of the bone defect size was based on the work of Kaban and Glowacki [17]. The soft tissue above the defect was closed with 4-0 Vicryl sutures (Ethicon, Lenneke Marelaan, Belgium). Eight surgical sessions were performed, and 12 of the operated rats suffered an intraoperative mandibular fracture and were sacrificed.

The postoperative course of the operations was uneventful, and all animals recovered normally, showing no signs of infection or other abnormalities, except for some para-mandibular swelling after ostectomy, which resolved spontaneously. At the conclusion of the experimental periods (3 and 6 months after implantation), the animals were euthanized by injection of an overdose of pentobarbitone sodium BP (100 mg/kg i.v.). Specimens were obtained by bisection of the mandibular symphysis, dislocation, and bloc hemimandibular excision.

#### 2.4.2. Histological Study

The experimental area was removed using an 8 mm trephine burr (Surgimedic, Asturias, Spain). They were fixed in 10% formalin (24 h, 4 °C) and macroscopically evaluated considering the following parameters: (a) anatomic and tissue organization of the defect, (b) infections, (c) biomaterial displacement or extrusion, (d) bone sequestration, and (e) consistency and morphology of the defect. Newly formed bone and osteoblastic cells were stained with hematoxylin and eosin (H&E). Samples were fixed for 5 days in 4% paraformaldehyde and then immersed in a graded ethanol series to dehydrate. The undecalcified bone specimens were infused and embedded in glycol-methylmethacrylate (GMMA; Technovit 9100, Kulzer, Germany). We obtained a sagittal section along the axis of the vascular canal of each implant using a precision saw (Isomet 1000; Buehler, Uzwill, Switzerland), and 5 μm serial sections were sectioned using a hard tissue microtome (Polycut SM 2500; Leica, Wetzlar, Germany). Samples were stained with H&E and examined with an optical microscope (Leica-DM 4000 B, Wetzlar, Germany).

#### 2.4.3. Radiological and Micro-CT Analyses (μCT)

**Figure 3:**
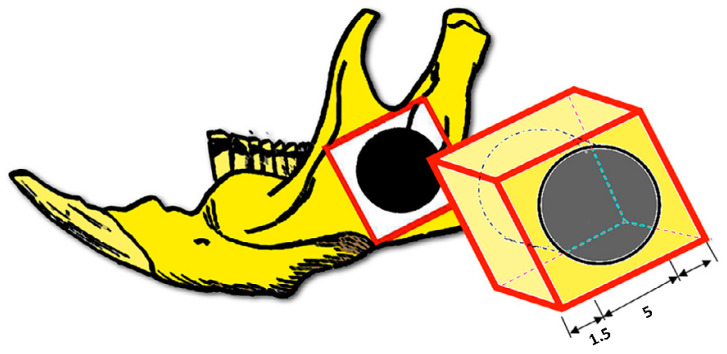
For micro-CT analysis, a square block is excised including the bone defect with a 1.5 mm bone margin.

For micro-CT analysis (μCT), two areas of interest were determined for each experimental right hemimandible: native bone (1.5 mm around the surgical defect) and new-formed bone (Figure 3). Qualitative analysis of the total mineral and newly formed bone contents was performed using a high-resolution micro-CT system (SkyScan 1174, SkyScan, Kontich, Belgium) and subjected to 3D reconstruction using NRecon software (SkyScan 1174, NRecon software, Bruker, Billerica, MA, USA). The scanner was provided with a 20 to 100 kV (10 W) X-ray source and an 11-megapixel X-ray detector. Each specimen was set on a support with the sagittal suture oriented parallel to the X-ray detector and scanned using a 0.11 mm copper filter, 26 μm isotropic voxels, a rotation step of 0.9°, and an average frame rate of 2. The following parameters were studied: bone mineral density (BMD), given in grams of hydroxyapatite per cubic centimeter (gHA/cm^3^), bone volume/total volume (BV/TV), defined as the ratio between the segmented bone volume and the total volume of the region of interest, measured as a percentage, trabecular separation (TbSp) expressed in mm, trabecular thickness (TbTh), expressed in mm and trabecular number (TbN), expressed in 1/mm. In summary, the values were compared between the native bone (around the generated defect) and the newly formed bone in the defect (Figure 3).

#### 2.4.4. Statistical Analysis

All statistical calculations were performed using SPSS software 27.0.1. (SPSS Inc., Chicago, IL, USA). Quantitative micro-CT parameters are expressed as mean ± standard deviation, unless otherwise stated. After testing for normality and equal variance, differences between native and new bone were analyzed in all rats of the groups 3 and 4 (paired *t*-test or Wilcoxon test). Differences were considered significant when *p* < 0.05. To compare the results obtained at 32 weeks with those obtained at 12 weeks, the Student’s *t* test was used, creating a relative variable in each individual, taking the native (resident) bone as a reference. Differences were considered statistically significant when *p* < 0.05.

## 3. Results

### 3.1. Design and Fabrication of Scaffolds

**Figure 4:**
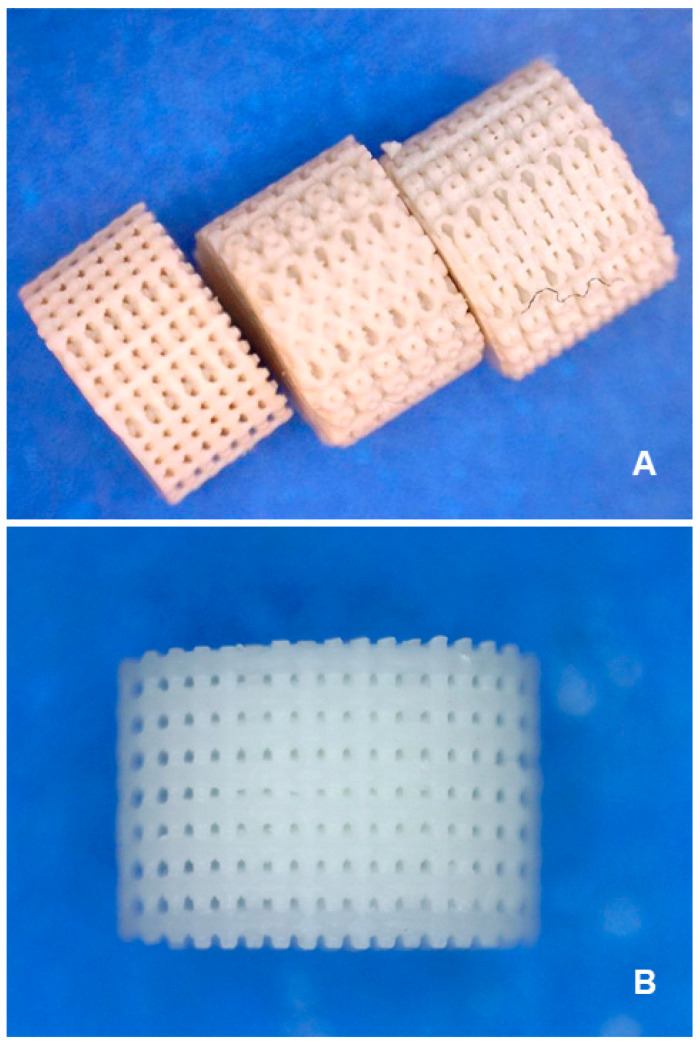
(**A**) Comparative resolution results on models A, B, and C, (from left to right). The D model was eliminated. (**B**) Model A’s final manufactured prototypes.

Out of the four proposed models (A–D), model D was discarded due to the impossibility of reproducing it with quality because of the small size of the connection channel (0.2 mm). After cleaning and printing the remaining three models (A–C) (Figure 4A), a comparison between them was carried out. The best results were obtained with model A (Figure 4B), which features internal holes, compared to B and C, where penetration was not possible even regarding the outermost wall. This is due to the arrangement of the spheres relative to the base piece and how the matrix is truncated at the beginning/end of the spheres. Therefore, exclusive fabrication of model A was undertaken, which consisted of spheres with intersections, aligned matrices, and a void volume of 24.52%. After being thoroughly cleaned, an analysis of the scaffolds at the green stage was performed to verify if they still retained material inside, along with destructive tests, cutting diametrical and height sections, to verify the existence of interconnection within the internal mesh. These tests revealed the presence of trapped material. Hence, a new cleaning process was carried out, applying an ultrasonic bath (at 37 KHz), extending the duration to three hours. Finally, the pieces were verified again and subjected to the thermal treatment process (sintering), aiming to have completely ceramic elements, free from the binding material. 

### 3.2. Mechanical Testing

To determine Young’s modulus, tensile strength curves were performed on two specimens of each of the scaffold models examined. All samples exhibited an initial zone of linear behavior (stress proportional to strain). From this linear zone, the Young’s modulus or the elastic modulus of the material can be obtained, E. This value provides the relation between strain and stress according to a linear relation of the type: σ = Eε, where σ is the stress, ε is the strain, and E represents Young’s modulus.

**Figure 5:**
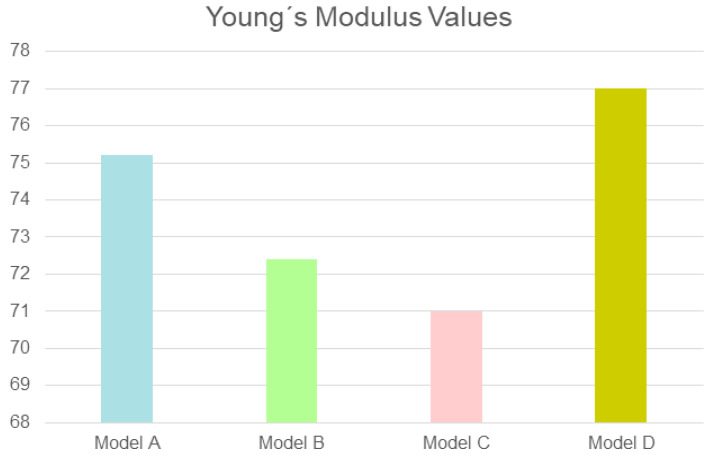
Young’s modulus values (Gpa) in the four models of this study. Model A: 75.2; model B: 72.4; model C: 71; model D: 77.

The tensile strength ranged between 285 and 295 MPa. The flexural strength ranged from 85 to 91 MPa. The mean value obtained for Young’s modulus was 73.4 GPa (range: 71–77) (Figure 5).

### 3.3. SEM Observations

**Figure 6:**
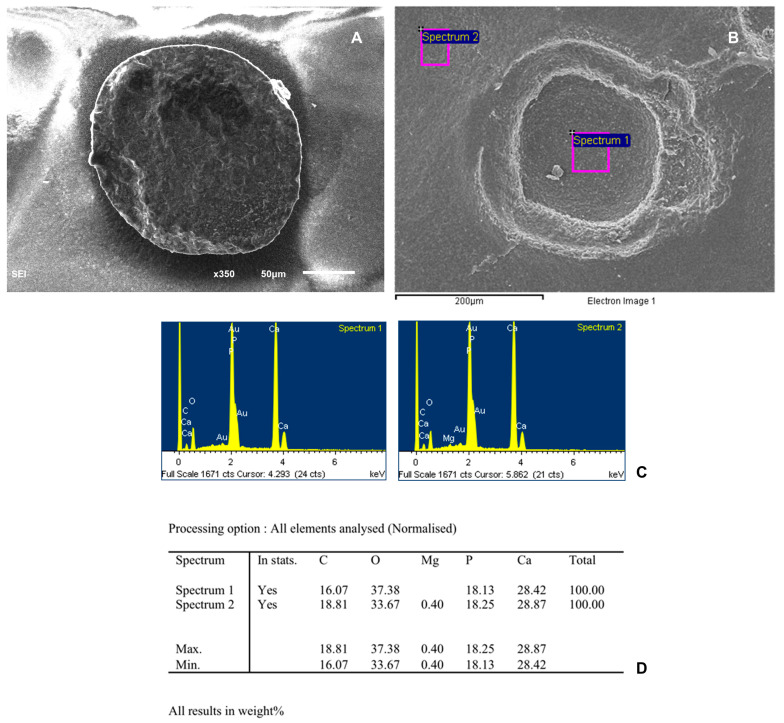
(**A**) SEM photographs of the scaffold without cells. (**B**) SEM image of scaffold showing the two points use for histomorphometric analysis. (**C**) Spectra of the different points analyzed. (**D**) Table showing percentages of elements analyzed.

**Figure 7:**
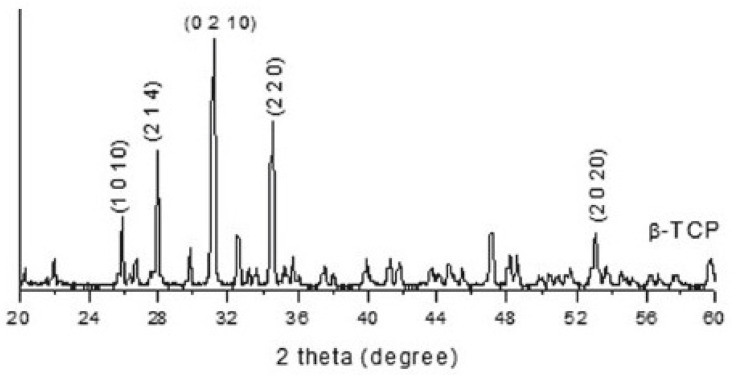
X-ray diffraction patterns. Particle size distribution of β beta-tricalcium phosphate powders in our scaffold.

SEM photographs of the scaffold without any type of cells are shown in Figure 6. Our scaffold has a solid structure and porous microstructure with a stable pore size of 500 microns. Almost all the peripheral pores were interconnected, but the central ones showed no interconnections. To confirm presence of b-TCP, the phases presented in our scaffold were also analyzed using a Siemens D5000 diffractometer, with CuKα1 radiation filtered with Ni, λ= 1.5406 Å, a time constant of 0.4, an angular step constant of 0.02, and operating at 30 Kv and 25 mA, over an angular range from 10° to 65° of 2Θ. The diffraction pattern of granules obtained from the TCP showed the mean peaks of β beta-tricalcium phosphate or whitlockite (JCPDS 090169) (Figure 7).

**Figure 8:**
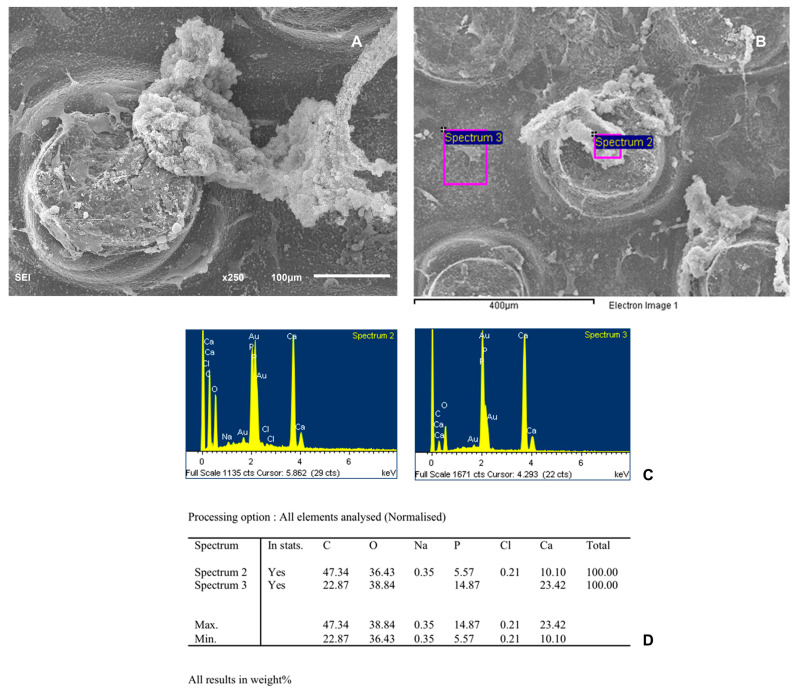
(**A**) SEM photographs of the osteogenic-differentiated cells cultured scaffold 7 days after plating showing a granular surface covered with cells sheets. (**B**) SEM image of scaffold showing the two points use for histomorphometric analysis. (**C**) Spectra of the different points analyzed. (**D**) Table showing percentages of elements analyzed.

**Figure 9:**
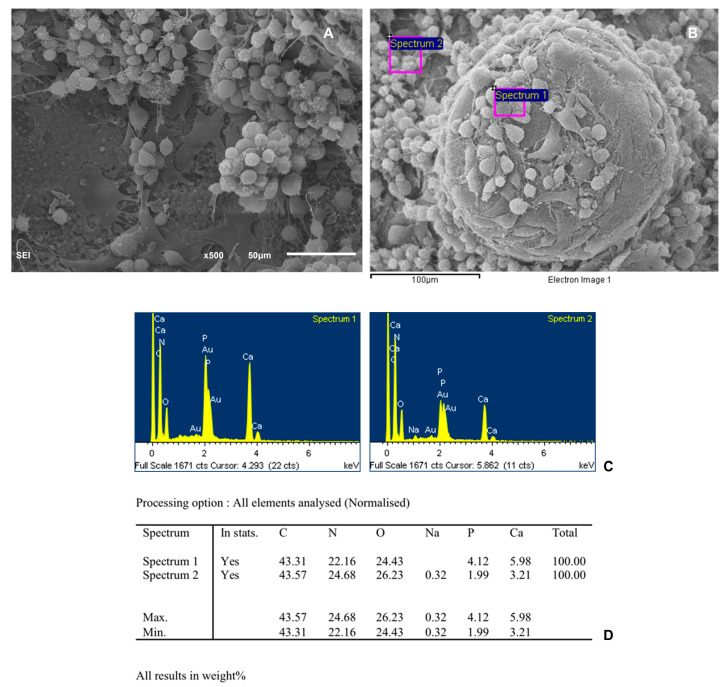
(**A**) SEM photographs of the undifferentiated cells cultured on scaffold 7 days after plating. (**B**) SEM image of scaffold showing the two points used for histomorphometric analysis. (**C**) Spectra of the different points analyzed. (**D**) Table showing percentages of elements analyzed.

SEMs photographs of cells cultured on the scaffold 7 days after plating show a granular surface covered with cell sheets. Figure 8 and Figure 9 show the percentage of elements analyzed at two different points of the scaffold, with undifferentiated cells and with differentiated cells, respectively. The porous three-dimensional structure of the scaffold provided intercellular contact and extracellular matrix accumulation. Cells adhered and sprouted their cytoplasmic process on the surface and grew well on the porous surface. Most of the particle surfaces were covered by a sheath-like complex of ECM and cells. Newly deposited minerals were also observed in the studied areas.

### 3.4. Early Detection of Osteogenesis

Alkaline phosphatase was detected in the AD-MSCsO scaffold cultures after 4 weeks of differentiation. However, the scaffolds alone or with AD-MSCs did not stain. 

### 3.5. In Vivo Results

Macroscopically, the consistency and morphology of the grafted area showed no remarkable alterations. None of the recovered jaws showed signs of infection or bone sequestration.

#### 3.5.1. Histological Analysis

**Figure 10:**
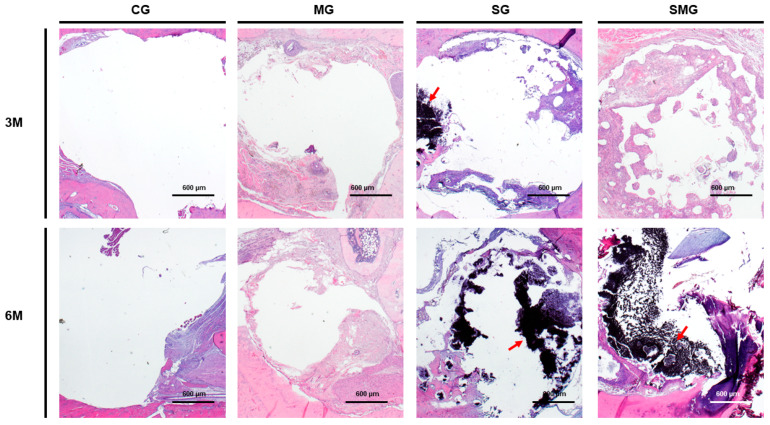
Histological evaluation of bone grafts stained with hematoxylin and eosin 3 months (3 M) and 6 months (6 M) after transplantation. No new bone formation was observed in any of control group (CG) animals. Minimal immature osteoid tissue surrounded by fibrous tissue is observed in membrane group (MG). Scaffold group (SG) samples showed immature newly formed bone surrounded by irregular osteoid tissue and non-degraded scaffold present at 3 and 6 months (arrow). Scaffold and membrane group (SMG) images demonstrating more mature and mineralized bone tissue and non-degraded scaffold (arrow).

The final sample comprised 30 animals for the histological study. Irrespective of the grafting time, no new bone formation was observed in any of the control animals. Quantitatively, in the membrane group (group M), bone formation was observed in two of the seven specimens analyzed. One of them corresponded to a rat sacrificed at three months, with a percentage of neoformed bone of 20%, and the other specimen was from a rat sacrificed at six months, with a percentage of neoformed bone of 40%. In the scaffold group (group S), the presence of new bone was observed in six of the eight animals. Two of them corresponded to the grafting period of 3 months with an average percentage of new bone formation of 25.5%, while four belonged to the animals sacrificed at 6 months, with an average percentage of bone formation of 28.2%. Finally, in the scaffold group with membrane (group SM), bone formation was achieved in five rats sacrificed at six months, with an average percentage of 28.2%, and in one of those sacrificed at 3 months (23.5%). In three animals, there was no evidence of bone formation. Figure 10 illustrates schematically the bone formation of the different groups under study. 

#### 3.5.2. Micro-CT Analysis

**Figure 11:**
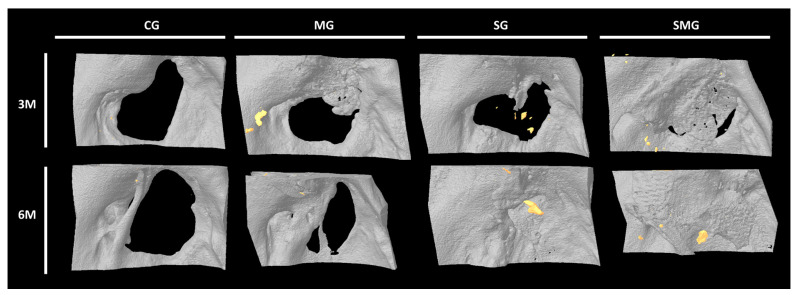
Representative micro-CT images of control transplanted animals at 3 months (3 M) and 6 months (6 M) post-operation. Control group (CG), shows no bone regeneration. Membrane group (MG) images show minor ossification located in the areas that faced native mandibular bone. Scaffold group (SG) and scaffold and membrane group (SMG) demonstrate a satisfactory defect regeneration at 6 months.

In none of the control defects was the formation of new bone evident, nor was it observed in some of the animals belonging to the membrane group, so it was not possible to homogeneously delimit the ROI in order to proceed with the automated analysis. Therefore, a qualitative analysis was carried out to indicate the absence or presence of newly formed bone (YES/NO) and to evaluate it from 1+ (vestiges) to 3+ (almost the entire surface) according to the surface it occupies. This information is collected in Table 1 and Figure 11.

In scaffold and scaffold and membrane groups (groups S and SM), the region of interest (ROI) was the area radiologically interpretable as bone by two experts. The quantitative values of this area were compared with the native bone of the same animal and with the grafting time (3 and 6 months).

Table 2 and Table 3 detail the values of the micro-CT variables in the rats of groups S and SM, regardless of the grafting time. In both groups, the BMD and BV/BT values were higher in the native bone than in the newly formed bone. However, this difference was only significant, in both groups, for the BV/BT variable and in group S for the trabecular number (Tb.N.).

Finally, the results obtained at 3 months were compared with those obtained at 6 months. The BMD variables and the BV/TV ratio showed higher values in the newly formed bone at 6 months. However, only BMD showed significant differences. 

## 4. Discussion

TCPs have been commonly used in reconstructive surgery since the 1980s, and many scientific papers have been reported on the subject so far. Most publications on this subject concern the treatment of the surrounding bone and therefore fall into the category of bone substitutes. However, the following directions of application of calcium phosphate in reconstructive surgery can be outlined: coverings of various types of dental implants, augmentation of surrounding tissues, and use as bone fillers of the jaw or other bones [8].

Over the last few years, the use of α- and β-TCP-based ceramics has become generalized, mainly as a result of the higher solubility of TCP in contact with body fluids [18]. The β-TCP exhibits lower solubility in water compared to α-TCP, which is more reactive in aqueous systems. When compared to HA, β-TCP has enhanced biodegradability and resorption rates, which may increase the biocompatibility of implants. The β-TCP has a lower resorption rate relative to α-TCP, and the nanoporous structure of β-TCP provides improved biomineralization, cell adhesion, and osteoblast proliferation [19].

LithaBone TCP 300 is a ceramic based on beta-tricalcium phosphate (ß-TCP). Tricalcium phosphate has a high degree of biocompatibility, bioresorbability, and osteoconductivity, making it a well-established material for bone substitute in regenerative medicine. Based on its properties, patient-specific resorbable implants with specific pore structures and geometries can be fabricated with this material. These implants will be reabsorbed by the body during the healing phase and replaced by native bone tissue, which means that a second surgical intervention to remove the implant is not necessary [8].

This study aimed to evaluate scaffolds’ fabrication using a 3D printing technology and assess their performance in bone regeneration through histological and μCT analyses. The chosen 3D printing technology, utilizing an LED light source and a grid screen, provided a viable alternative to traditional stereolithography. Model A, characterized by internal holes and a void volume of 24.52%, demonstrated superior results compared to other designs, emphasizing the significance of scaffold design for effective bone regeneration. Studies with our experimental animals showed good osseointegration results with relatively simple geometries, avoiding the use of trabecular models that require more complex computational and manufacturing difficulties.

Three-dimensional-printable TCP in LCM technology offers an interesting alternative as a bone grafting material.

Meshed structures, whose fabrication has been made possible by additive technologies, are increasingly gaining popularity in all fields where lightness is an imperative. Because the complexity of lattice geometries exceeds the technological limitations of even additive processes, the fabricated structures can differ significantly from the nominal ones, not only in terms of expected dimensions but also in the number of defects. Consequently, the successful use of lattices demands the combined design optimization, structural modeling, construction orientation, and configuration [20].

### 4.1. Fabrication

Conventional methods of manufacturing porous scaffolds include foam rendering, solvent casting, and freeze-drying [21]. These methods provide limited control over scaffold chemistry, macrostructure, and porosity. Alternative methods have been proposed to solve the problems of scalability, sustainability, and spatial control [22].

Manufacturing techniques used for the fabrication of bone scaffolds can be classified, depending on the method of fabrication, into subtraction and addition. In addition, depending on the level of manual versus computer control in the design and fabrication process, the techniques can also be categorized as conventional (less computerized) or current techniques (more computerized). Conventional techniques had the general problem that the pore architecture cannot be customized, resulting in considerable difficulty in controlling the size of the pores as well as in obtaining their controlled interconnection. According to Thavornyutikarn et al. [23], these conventional techniques are largely unable to produce a completely continuous interconnectivity and uniformed pore morphology within a scaffold. Most conventional techniques produce pores by subtraction. On the other hand, current additive manufacturing techniques, also called solid free-form fabrication techniques, offer the ability to individualize scaffolds and generate complex geometries with precisely controlled porosity [24].

In a recent systematic review of the literature that aimed to evaluate existing methods to fabricate personalized bone scaffolds through 3D technology, 52 articles describing 14 techniques (4 subtraction techniques + 10 addition techniques) were identified [25]. 

Additive manufacturing has paved the way for patient specific biomaterials, and holds much promise within the orthopedic and maxillofacial implant fields [26,27]. It also provides advantages in scale, cost, and flexibility over conventional manufacturing methods. Seven different additive manufacturing processes based on deposition and bonding have been classified by the American Society for Testing and Materials: photopolymer vat, material jetting, material extrusion, powder bed infusion, directed energy deposition, foil lamination, and binder jetting [28]. All of these methods have been applied in biomedical engineering applications [8,29].

The methodology developed in our laboratory for the manufacture of the scaffold presents two important drawbacks. On the one hand, the limited computational capacity associated with the parametric software made it impossible for us to reproduce these meshes with pore diameters of 0.2 mm (too many elements), as well as extrapolate it to another geometry. On the other hand, although we confirmed an interconnection between the channels closest to the peripheral wall, we were also able to verify a clear collapse in the core of the cylinder. Lengthening the ultrasonic cleaning cycle improved this last limitation, but, as reflected in the SEM images, a limit to the improvement was identified, independent of the residence time. For this reason, and in our opinion, the biggest obstacle in the manufacturing phase of bone implants using LCM technology is found in the post-processing and cleaning phase.

The long-standing dogma of optimal mean pore size is mostly related to observations made on scaffolds with either single channels or randomly distributed pores. Since then, the development of additive manufacturing has introduced a new dimension to the fabrication of scaffolds. In this method, pore size and other microarchitectural constraints, such as bottleneck dimensions, can be precisely defined [8]. In this context, the bottleneck dimension is defined as the uniform diameter of the pore-to-pore connections and can be exactly adjusted by additive manufacturing. In random pore distribution processes, the term percolation diameter is defined as the diameter of the largest tracer sphere capable of moving through a scaffold of interconnected pores and reflects the smallest diameter of a single connection in a system of interconnected pores [8]. Pore interconnectivity is associated with rapid bone regeneration, vascularization, and material resorption [30].

### 4.2. Mechanical Testing and In Vitro Study

The optimal bone substitute should supply biomechanical support by creating a favorable microenvironment for cells to attach, proliferate, and differentiate, thereby leading to bone ingrowth and creep replacement, the main characteristics of osteoconduction. Currently, the concept of the triply periodic minimal surface (TPMS) designs is being transferred to bone matrices as an interesting option. TPMSs are infinite surfaces with periodicity in three dimensions, with no self-intersections and zero mean curvature [31].

The main reason for this growing interest is that their surface tortuosity is analogous to that of trabecular bone. In addition, these continuous surfaces with smooth junctions result in lower stress concentrations and correspond to superior mechanical characteristics, lead to improved cell adhesion and proliferation, and thus to increased bone tissue ingrowth [32]. The compressive strengths of β-TCP scaffolds range from 1.4 ± 0.5 MPa to 67.6 ± 13.3 MPa over a porosity range of 5.58 ± 0.09% to 59.36 ± 0.18%. The strength of these scaffolds is lower than the compressive strength of cortical bone (100–200 MPa), but similar to the compressive strength of cancellous bone (0.1–16 MPa). It is widely accepted that the mechanical performance of a composite material depends on several factors, such as the aspect ratio of the reinforcing agent, the degree of dispersion of the filler in the matrix, and the adhesion at the filler-matrix interface. For example, tricalcium phosphate and magnesium oxide composites were characterized using physical properties such as fracture toughness, Vickers hardness, and elastic modulus. The highest mechanical strength and Young’s modulus of the composites reached 9 MPa and 38 GPa, respectively [32,33,34,35].

Many different types of bones are present in the human body, and all of them have varying mechanical properties from each other. Consequently, it is not feasible to prepare a suitable bone scaffold for all bones. The cortical bone and the trabecular bone, for example, have a tensile strength of 172 and 1.6 MPa, respectively. Young’s modulus is highly correlated with the stiffness of the material [36].

Misch et al. [37] performed compression tests using cadaveric cylindrical specimens to determine the Young’s modulus of the human trabecular mandibular bone. The samples were drilled from the bone in a vertical direction and frozen in storage. Cortical layer-covered specimens gave a Young’s modulus of 24.9–240 MPa (mean: 96.2 MPa, standard variation: 40.6 MPa), while those with a machined surface gave 3.5–125.6 MPa (mean: 56.0 MPa, standard variation: 29.6 MPa). Other authors report similar results [36].

In our study, the assessment of mechanical properties indicated that the values of technical ceramic for 3D printing are at the maximum for bone compression and triple the maximum for the elasticity modulus, indicating suitability for maxillofacial use. The compression and flexion resistance values of ceramic elements developed by LCM technology are crucial for potential maxillofacial applications, where loads have a predominant compression component during the chewing process. As the rod width of the printed scaffolds increases, the porosity decreases and the compression modulus and elasticity change. Therefore, it can be concluded that our matrices have adequate tensile strength and stiffness to use as a bone scaffolds.

In vitro, osteoblasts could grow and maintain their secretory activity when cultured on the investigated scaffold in the present study. Regarding early osteogenesis detection, alkaline phosphatase identification in AD-MSCsO scaffold cultures after 4 weeks of differentiation is a crucial finding. This suggests that the manufactured scaffolds support cellular activities indicative of initial bone formation, supporting the potential utility of 3D-printed scaffolds in bone tissue engineering applications. Often, the number of endogenous cells capable of migrating to a scaffold and differentiating into the desired tissue is not sufficient to achieve complete regeneration. For this reason, classical tissue engineering focuses on the use of autologous cells expanded in culture and finally reimplanted in the patient, as the main facilitating mechanism for complete regeneration. This combination can be used with our scaffold [38,39].

### 4.3. In Vivo

The use of 3D-printed hydroxyapatite scaffolds for stimulating bone healing has been increasing over the years. However, in the last 20 years, only 20 studies have been recognized in the literature in which its behavior in animals was evaluated: 13 studies were performed using New Zealand White rabbits and seven papers used Sprague-Dawley rats. In addition, 10 studies performed the bone defect in the animals’ legs: one in the femur, one in the tibia, two in the radius, and six in the femoral condyle. In addition, 10 studies used the calvarial bone defect model. Regarding the studies in which rats were used, two studies used 12-week-old animals, and five did not specify the age of the animal. The weight of the rats ranged from 180 g to 350 g. Defect sizes ranged from 3.5 mm to 15 mm in diameter [40].

For the present study we have used 42 adult female Wistar rats as experimental animals. The realization of critical mandibular defects in these small animals presented two important limitations. On the one hand, the small mandibular thickness minimizes the graft–native bone contact surface, and on the other hand, the homogeneous preparation (with a trephine) of the 5 mm critical defect caused a high number of mandibular fractures (12 animals). The reasons for selecting this animal model were economic, ethical, and statistical. However, in our opinion, Wistar rats are not the most appropriate model for the replication of a scenario that attempts to evaluate mandibular osteopromotion.

To our knowledge, there are few clinical trials evaluating the use of bone scaffolds. In the review by Crowley et al. [41], only five studies on scaffolds for bone regeneration are related to clinical trials. In them, three of these studies were related to small numbers, four of the studies did not have a control group, and all the studies involved a short follow-up time of several months and even weeks. However, of the 20 articles included in Zeng et al.’s [42] review, only four studies used small samples of less than 10 specimens. More than half of them included one or even more than one control group. The follow-up time also increased from a few months to more than 1 year in most studies. This review included twelve clinical studies on synthetic structures with Hydroxyapatite, β-TCP, and their complexes to repair bone defects. In this systematic review, most of the works were related to femoral and acetabular defects (four studies), or to tibial fractures (three studies). All of them reported positive results for clinical bone regeneration.

In vivo, the image analysis most used in the different works was micro-CT. Different authors observed that the scaffolds produced an increase in the deposit of newly formed bone in the area of the defect and greater bone volume [40].

Although some authors [43] demonstrated that rat calvarial defects filled with β-TCP presented the highest BV/TV value in relation to the PLA/HA, our results are not as satisfactory. However, in the study by Tu et al. [44], similar results to those of the present work were obtained: BV/TV of approximately 10%, 4 weeks after surgery.

In our work, the experimental groups (groups S and SM: TCP alone or with membrane, respectively) showed a higher regenerative capacity than the control group (critical defect: no bone formation) and group M, in which only membrane without scaffold was used. In this M group, we could only observe slight bone formation in the animals sacrificed at 6 months.

The guided bone regeneration (GBR) procedure is based on the use of a membrane that is applied between the surrounding soft tissue and the bone defect [45,46]. This membrane acts as a barrier and prevents the invasion of soft tissue in the area of the bone defect, thus allowing osteoprogenitor cells to preferentially fill the bone defect, allowing for bone regeneration [47]. Two main types of GBR membranes can be used clinically: non-resorbable and resorbable membranes. Despite the wide variety of barrier membranes available on the market, they all have limitations. The four requirements that Wang et al. [48] described to consider a GBR as successful are known, as the “PASS” principles: (1) primary closure of the wound, (2) angiogenesis for adequate blood supply to the defect, (3) maintenance of space of the newly formed bone, and (4) stability of the wound to allow the formation of blood clots. In this group of animals in our study, bone neoformation was minimal in the animals sacrificed at the third month and slightly greater in those that were euthanized at 6 months. 

In this work, we have studied the regenerated bone of groups 3 and 4, comparing the characteristics of the newly formed bone with those of the neighboring native bone using micro-CT. The BV/TV ratio was significantly higher in native bone than in newly formed bone, but BMD did not show statistical differences. Furthermore, although the volume of newly formed bone was greater in the animals sacrificed at 6 months, this difference did not reach statistical significance with the 3-month group. However, at 3 months, the BMD values were significantly lower. In summary, the volume of newly formed bone is lower than that of the native bone, but its maturity is very similar. 

Quantitative μCT analysis supported the histological observations. The formation of new bone was greater in the animals in the scaffold group and the scaffold and membrane group, slightly higher in the latter than in the membrane and control groups. Surprisingly, although a longer graft time was accompanied by a greater amount of newly formed bone, the differences between the two sacrifice times were not significant. 

In essence, histological analysis of the β-TCP cylinders implanted in rats revealed varying degrees of bone formation across different groups. The membrane-only group showed limited bone formation, while group S, treated with a graft, exhibited improved regenerative outcomes. Group SM (treated with both membrane and graft) demonstrated notable bone formation, suggesting that the incorporation of a graft enhances bone regeneration. The possible clinical relevance of 3D printed scaffolds in promoting bone regeneration is suggested by the differences observed between the groups, which emphasizes the importance of considering both the presence of a graft and the use of a membrane in the design of the scaffolds. More research is needed, especially in larger animal models and clinical trials, but this study provides a promising foundation for advanced 3D printed structures in bone tissue engineering.

At present, special attention has been directed to the design of new scaffolds through the addition of bioactive molecules and nanoparticles [14,15]. In bone tissue engineering, “smart scaffolds” not only act as cell-delivery materials, but also react to their environment, and thus stem cells are more likely to adhere, proliferate, and differentiate. These scaffolds can be produced by adding growth factors, extracellular matrix proteins, or nanoparticles to bone substitutes using a diversity of techniques. Such adaptations can improve the in vitro response of bone scaffolds to cells [49].

In summary, tissue engineering has emerged as a novel approach to repair bone defects. This study was carried out to investigate the effectiveness of additive manufacturing, specifically three-dimensional printing, of β beta-tricalcium phosphate scaffolds for mandibular bone regeneration in critical jaw defects of rats. Its main finding was that bone substitutes constructed according to the investigated methodology can repair critical defects in rats but incompletely, despite the maturity of the neoformed bone. The final goal of this study is the clinical application of the experimental scaffold in bone defects of the jaws secondary to traumatic or oncologic pathologies or related to bone resorption associated with tooth loss. Although the cylindrical defect investigated in rats is comparable to mandibular defects caused by odontogenic cysts or tumors, other defects requiring more complex treatment could also be approached with the described methodology. 

The practical implications for surgeons are that the product investigated could be considered a cheap scaffold, quick to manufacture, and suitable for CAD-CAM guided bone regeneration. For cost purposes, the integrated process of manufacturing an implant using this technology, considering hours of design/engineering, manufacturing, and even testing on printed models, would be around €50–100 with a delivery time of 12–15 days. 

## 5. Conclusions

The integration of innovative 3D printing technology, meticulous scaffold design, and effective cleaning processes holds promise for enhancing bone regeneration. This study’s comprehensive evaluation through histological and μCT analyses provides valuable insights into the potential clinical applicability of 3D-printed scaffolds. Future research should focus on refining fabrication techniques and conducting long-term clinical studies to establish the safety and efficacy of these scaffolds in human patients.

## Figures and Tables

**Table 1:** Control and membrane groups. Qualitative results of the newly formed bone and correlation with healing time: + (vestiges); ++ (almost half the surface); +++ (almost the entire surface). * Peripheral bone growth, with slight decrease in the initial defect.

Membrane	Healing Time (Months)	New Bone	Control	Healing Time (Months)	New Bone
1 M	3	Yes (+)	1 C	3	No
2 M	3	Yes (+)	2 C	3	No
3 M	3	Yes (+)	3 C	3	No
4 M	3	No	4 C	6	No *
5 M	6	Yes (+++)	5 C	6	No *
6 M	6	Yes (+++)	6 C	6	No *
7 M	6	Yes (++)			
8 M	6	Yes (++)			

**Table 2:** Micro-CT results in scaffold group (S). BMD, bone mineral density; BV/TV, bone volume/total volume; TbTh, trabecular thickness; TbSp, trabecular separation; TbN, trabecular number.

Group S		Mean	Standard Deviation	Mean Differences	Sig. (Bilateral)
**N = 8**	**BMD. native**	0.919	0.110	0.417	0.096
**N = 8**	**BMD. new**	0.790	0.502		
**N = 8**	**BV.TV. native**	70.04	12.33	43.15	**0.001**
**N = 8**	**BV.TV. new**	26.89	7.367		
**N = 8**	**Tb.Th. native**	0.153	0.036	0.03	0.077
**N = 8**	**Tb.Th. new**	0.123	0.048		
**N = 8**	**Tb.Sp. native**	0.119	0.069	−0.303	0.07
**N = 8**	**Tb.Sp. new**	0.422	0.268		
**N = 8**	**Tb.N. native**	5.06	0.130	4.162	**0.03**
**N = 8**	**Tb.N. new**	0.898	0.427		

**Table 3:** Micro-CT results in scaffold with membrane group (SM). BMD, bone mineral density; BV/TV, bone volume/total volume; TbTh, trabecular thickness; TbSp, trabecular separation; TbN, trabecular number.

Group SM		Mean	Standard Deviation	Mean Differences	Sig. (Bilateral)
**N = 8**	**BMD. native**	0.917	0.202	0.018	0.1
**N = 8**	**BMD. new**	0.899	0.321		
**N = 8**	**BV.TV. native**	77.04	10.52	50.59	**0.001**
**N = 8**	**BV.TV. new**	26.45	8.46		
**N = 8**	**Tb.Th. native**	0.192	0.044	0.01	0.087
**N = 8**	**Tb.Th. new**	0.183	0.043		
**N = 8**	**Tb.Sp. native**	0.125	0.044	−0.067	0.08
**N = 8**	**Tb.Sp. new**	0.192	0.135		
**N = 8**	**Tb.N. native**	5.8	0.151	1.5	0.089
**N = 8**	**Tb.N. new**	4.3	0.503		

## Data Availability

The data that support the findings of this study are available from the corresponding author upon reasonable request.
